# Novel insight into the correlation between hernia orifice of cystocele and lower urinary tract function: a pilot study

**DOI:** 10.1186/s12905-022-01747-5

**Published:** 2022-05-13

**Authors:** Takeya Kitta, Hirokazu Abe, Huang Ting-wen, Masahiro Fujikawa, Minoru Nakazono, Taiki Sasa, Yukiko Doi, Sari Toki, Daigo Okada, Atsuhiko Ochi, Koichiro Suzuki, Yasuhide Kitagawa, Nobuo Shinohara

**Affiliations:** 1 Department of Renal and Genitourinary Surgery, Graduate School of Medicine, Hokkaido University, Kita 15 Nishi 7; Kita-ku, Sapporo, Hokkaido 060-8638 Japan; 2 Department of Urology, Kameda Medical Center, Kamogawa, Japan; 3 Department of Urology, Ushikuaiwa General Hospital, Ushiku, Japan; 4 Department of Urology, Komatsu Municipal Hospital, Komatsu, Japan

**Keywords:** Pelvic organ prolapse, Voiding dysfunction, Anterior wall prolapse, Cystoscopy, Quality of life

## Abstract

**Background:**

It has been hypothesized that women with significant pelvic organ prolapse (POP), particularly of the anterior vaginal wall, may have voiding dysfunction (VD). Although the VD mechanism due to cystocele is not fully understood, different vaginal compartments have rarely been closely examined. This study attempted to further elucidate the correlation between POP and VD through a new subgroup classification using cystoscopy.

**Methods:**

This study reviewed clinical records of 49 women who underwent cystocele repair. All patients were scheduled for laparoscopic sacrocolpopexy, preoperatively underwent uroflowmetry and postvoid residual urine volume (PVR) measurement, and completed pelvic floor function questionnaires. Bladder examination by cystoscopy was additionally performed using the lithotomy position with the Valsalva maneuver.

**Results:**

Subjects were divided into four groups according to hernia orifice presence determined by cystoscopy, which included the trigone type, posterior wall type, trigone and urethra type, and trigone and posterior wall type. The posterior wall type had statistically higher PVR values versus the trigone and posterior wall type (*P* = 0.013). The posterior wall type had statistically lower values for average urine flow rate versus the urethra and trigone type (*P* = 0.020). There were no significant differences noted in the pelvic floor function questionnaires among the four groups.

**Conclusions:**

A new bladder defect classification based upon hernia orifice location was associated with lower urinary tract function. Posterior wall hernia presence caused significant voiding function deterioration. This new subgroup classification, which can more clearly identify and indicate bladder function, is also comparable among patients.

**Supplementary Information:**

The online version contains supplementary material available at 10.1186/s12905-022-01747-5.

## Background

The prevalence of pelvic organ prolapse (POP) is increasing among aging women and thus, is likely to become a common condition worldwide. Women have an 11% lifetime risk of surgery for urinary incontinence or POP [1]. Thus, POP needs to be considered as one of the more significant medical issues in our society. Moreover, cystocele is the most commonly seen among all of the conditions associated with POP. High-grade cystocele is generally associated with lower urinary tract dysfunction, such as voiding dysfunction (VD) and stress urinary incontinence (SUI) [2, 3]. However, the mechanism of lower urinary tract dysfunction due to cystocele has yet to be fully understood. Moreover, there have yet to be any definitive studies that have examined the correlation between POP and lower urinary tract dysfunction, especially with regard to the anatomical changes of cystocele. Moreover, the different bladder compartments have rarely been closely inspected for lower urinary tract dysfunction in the POP patient. This current study attempted to further clarify the impact of anatomical changes on bladders with cystocele and the subsequent changes in the lower urinary tract function.

The primary objective of the current study was to develop a new clinical classification based upon both the position and the number of bladder hernia orifices of cystocele that were determined via the use of cystoscopy. Subsequently, we then evaluated this new classification and its association with objective and subjective symptoms.

## Methods

**Fig. 1:**
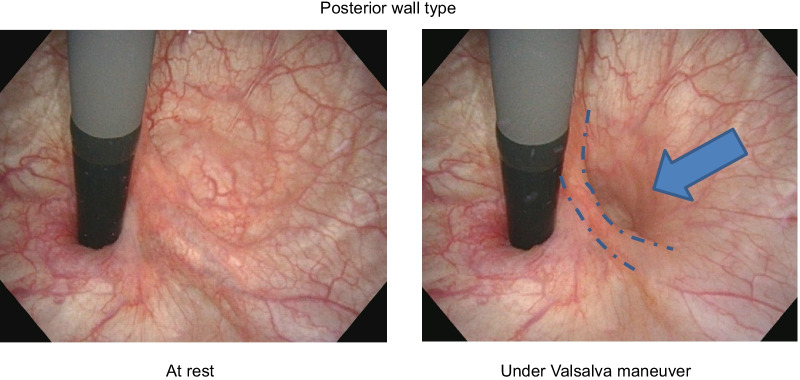
Bladder examination using cystoscopy was performed in the lithotomy position at rest and under the Valsalva maneuver. Dotted line: interuretieric crest. Arrow: hernia orifice

**Fig. 2:**
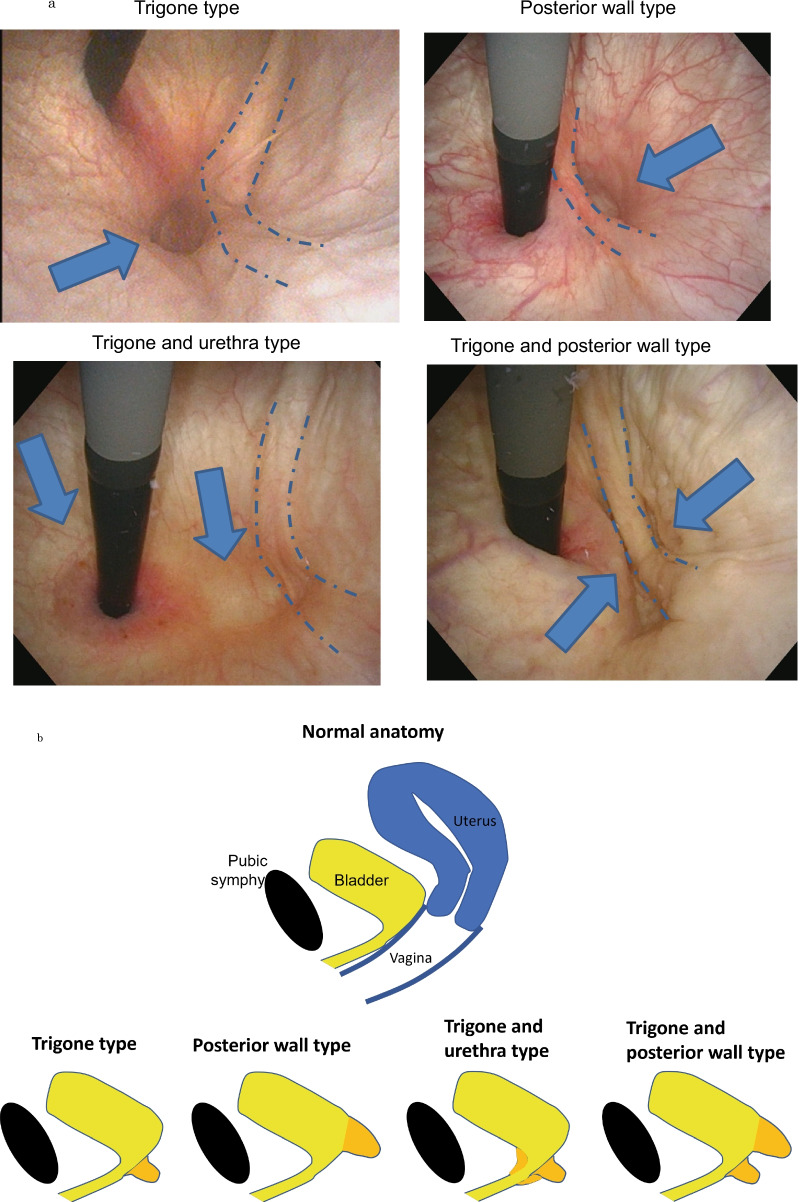
**a** The subjects were divided into four groups according to the presence of hernia orifice by cystoscopy: trigone type, posterior wall type, trigone and urethra type, and trigone and posterior wall type. Dotted line: interuretieric crest. Arrow: hernia orifice. **b** Schematic of four groups according to the presence of hernia orifice by cystoscopy: trigone type, posterior wall type, trigone and urethra type, and trigone and posterior wall type

This study evaluated 49 POP patients with high-grade cystocele (≥ POP-Q stage III) who underwent a preoperative office examination at our institute between 2018 and 2020. All women were scheduled for laparoscopic sacrocolpopexy while under general anesthesia. After Institutional Review Board approval, we reviewed a series of consecutive women who also had an available preoperative assessment. Sociodemographic, anthropometric measures, clinical data, gynecological and obstetrical history were collected in all subjects. The preoperative office examination was performed by the same physician in all cases (HA). To measure the postvoid residual urine volume (PVR), every patient underwent preoperative free uroflowmetry and ultrasound after voiding. The degree of POP was staged according to the POP-Q system. Additionally, bladder examination using cystoscopy was performed with the Valsalva maneuver. The detailed method is explained below. Patient should empty her bladder before the procedure. Patient is positioned on the examination table (frog-leg supine) (Fig. 1). Begin the inspection of the bladder at the 12 o'clock position, withdraw the cystoscope enough to see the bladder neck, and then advance again while beginning the inspection at the one o'clock position taking in the entire arc of the bladder from the posterior wall to the bladder neck checking malignance or stone formation. During systematically survey, the trigone and interureteric ridge are identified just inside the bladder neck. Return the cystoscope to the 12 o'clock position, advance the cystoscope and completely retroflex the cystoscope from this position to inspect the bladder neck. The examiner's wrist is then rotated both clockwise and counterclockwise with the endoscope retroflexed to fully inspect the bladder neck and assess for the presence of herniation. Identify three types of hernias, such as urethra, trigone, and posterior wall type. The site is determined as follows (Fig. 2a). Herniation of urethra is herniated around the cystoscope. Herniation of trigone is positioned not around the cystoscope, but just foreground the interureteric ridge. Herniation of posterior wall is positioned just posterior the interureteric ridge. Classification was performed by these hernia orifice sites combination.

The pelvic floor distress inventory short form (PFDI-20) and international consultation of incontinence questionnaire-short form (ICIQ-SF) questionnaires were used for measurements of the clinic condition of the pelvic floor related to the symptoms perceived by the participants. The PFDI-20 [4] was used as a condition-specific questionnaire for prolapse (PFDI-20—Additional file 2: Appendix 1). The ICIQ-SF questionnaire is commonly used for evaluating the frequency, severity, and impact on quality of life of urinary incontinence in men and women in research and clinical practice throughout the world [5]. This study is non-funded. Our group conducted research without obtaining a license both PFDI-20 and ICIQ-SF because both questionnaires are in the public domain. Exclusion criteria included urinary tract malignancy, neurological disorder, cardiac diseases, inability to perform the Valsalva maneuver, and urinary tract infection during the evaluation period. Statistical analyses were calculated using the GraphPad Prism version 8.4.2 software package. Statistical analyses were performed using independent t-tests and a one-way analysis of variance with Tukey's multiple comparisons test (post hoc analyses). A *P* value less than 0.05 was considered to be statistically significant.

## Results

In 49 women, (age ranging from 50 to 91 years; mean, 72 years), the mean number of vaginal deliveries was 2 (range, 0–5) and the body mass index was 24.0 (range, 17.3–32.2). Table 1 presents the demographic characteristics for the patients of all types. The subjects were divided into 4 groups based on the presence of hernia orifice due to cystocele. The 4 groups were the trigone type, posterior wall type, trigone and urethra type, and trigone and posterior wall type (Fig. 2a, b and Table 1). Stage IV distribution in the POP-Q stage was only observed in the trigone and urethra type, and the trigone and posterior wall type (Table 1). There was a significant difference noted among the four groups for the point genital hiatus (Gh), which is the distance between the urethral meatus and the posterior hymen (*P* = 0.03). POP-Q point Gh was found to exhibit a greater value in patients with the trigone type versus the posterior wall type (4.9 ± 0.8 vs. 3.6 ± 0.5, *P* = 0.02) (Table 2). There was a significant difference noted among the four groups for the PVR (*P* = 0.010). The posterior wall type had statistically higher values for the PVR as compared to the trigone and posterior wall type (150.0 ± 133.2 vs. 22.4 ± 48.4, *P* = 0.01) (Table 3). There was a significant difference among the four groups for the maximum urinary flow rate. However, there was also a significant difference among the four groups for the average urinary flow rate (*P* = 0.02). The posterior wall type had statistically lower values for the average urinary flow rate as compared to the urethra and trigone type (7.9 ± 5.8 vs. 15.7 ± 6.6, *P* = 0.020) (Table 3). There was no significant difference observed among the four groups for both the PFDI-20 and ICIQ-SF (Table 4).

**Table 1:** Population characteristics

Characteristics	All patients	Trigone type	Posterior wall type	Trigone and urethra type	Trigone and posterior wall type
Number	49	7	10	13	19
Age (years)	72 (50–91)	73 (70–81)	71 (61–81)	71 (50–85)	73 (59–91)
BMI (kg/m^2^)	24.0 ± 3.4	24.5 ± 1.5	24.5 ± 3.2	23.9 ± 3.4	23.7 ± 4.0
Parity (median (range))	2 (0–5)	3 (0–3)	2 (2–3)	2 (2–3)	2 (1–4)
POP-Q stage III, IV	42, 7	7, 0	10, 0	12, 1	13, 6

Data are mean ± standard deviation or n (range)

*BMI* body mass index, *POP-Q* pelvic organ prolapse quantification

**Table 2:** Preoperative pelvic organ prolapse quantification (POP-Q)

POP‐Q point	Trigone type	Posterior wall type	Trigone and urethra type	Trigone and posterior wall type	*P* value	
Aa	2.6 ± 1.1	2.1 ± 1.3	1.8 ± 1.5	1.5 ± 1.4	0.39	
Ba	4.0 ± 1.3	4.1 ± 0.9	2.8 ± 1.4	3.7 ± 2.8	0.40	
C	2.9 ± 2.4	2.4 ± 2.2	1.2 ± 2.4	2.9 ± 3.4	0.43	
Gh	4.9 ± 0.8	3.6 ± 0.5	3.9 ± 0.7	4.1 ± 1.0	0.03*	T vs. *P* *p *= 0.02
Pb	2.5 ± 0.5	3.0 ± 0.2	2.9 ± 0.4	2.9 ± 0.3	0.09	
TVL	6.7 ± 3.6	7.8 ± 0.4	8.4 ± 1.1	8.1 ± 0.9	0.19	
Ap	− 0.4 ± 1.7	− 0.9 ± 1.5	− 1.4 ± 1.3	− 0.9 ± 2.0	0.70	
Bp	0.1 ± 1.4	0.7 ± 1.7	− 0.8 ± 2.4	1.5 ± 3.4	0.17	
D	− 2.1 ± 1.2	− 1.1 ± 2.5	− 3.1 ± 3.3	− 0.5 ± 4.4	0.24	

Data are mean ± standard deviation

*T* trigone type, *P* posterior wall type, * < 0.05

**Table 3:** Lower urinary tract function of four groups

Characteristics	Trigone type	Posterior wall type	Trigone and urethra type	Trigone and posterior wall type	*P* value	
Voided volume	275.4 ± 127.8	255.0 ± 171.4	395.8 ± 114.9	356.8 ± 118.7	0.07	
Postvoid residual urine volume	116.6 ± 104.0	150.0 ± 133.2	39.8 ± 113.7	22.4 ± 48.4	0.01*	*P* versus T&P *P* = 0.01
Maximal urinary flow rates	16.7 ± 7.6	14.9 ± 11.2	25.5 ± 10.9	23.1 ± 11.0	0.27	
Average urine flow rate	8.8 ± 3.7	7.9 ± 5.8	15.7 ± 6.6	11.5 ± 5.8	0.02*	*P* versus T&U *P* = 0.02

Data are mean ± standard deviation

*P* posterior wall type, *T&P* trigone and posterior wall type, *T&U* trigone and urethra type, * < 0.05

**Table 4:** PFDI-20 and ICIQ-SF score of four groups

Variables	Trigone type	Posterior wall type	Trigone and urethra type	Trigone and posterior wall type	*P* value
PFDI-20					
POPDI	11.2 ± 8.6	14.0 ± 5.1	11.2 ± 6.1	8.5 ± 8.0	0.38
CRADI	5.0 ± 6.2	8.4 ± 6.4	4.2 ± 5.1	4.2 ± 4.3	0.31
UDI	10.2 ± 5.4	11.7 ± 5.7	4.2 ± 4.3	7.1 ± 6.2	0.37
Total	26.3 ± 14.9	32.6 ± 14.6	25.5 ± 14.2	22.8 ± 15.4	0.56
ICIQ-SF					
Q1	2.8 ± 1.6	1.9 ± 1.6	0.9 ± 1.1	1.5 ± 1.5	0.10
Q2	1.6 ± 0.8	1.8 ± 1.5	1.2 ± 1.0	1.3 ± 1.2	0.69
Q3	1.0 ± 1.6	3.3 ± 2.8	2.3 ± 2.1	3.2 ± 3.5	0.44
Total	5.4 ± 0.5	7.0 ± 5.6	4.4 ± 3.7	5.9 ± 5.7	0.69

Scores of the PFDI-20 and ICIQ-SF score of four groups (n = 49)

*PFDI-20* pelvic floor distress inventory short form, *ICIQ-SF* international consultation of incontinence questionnaire-short form. The PFDI score is the total of the Pelvic Organ Prolapse Distress Inventory (POPDI) score (bulging), Colorectal-Anal Distress Inventory (CRADI) score (defecation), and Urinary Distress Inventory (UDI) score (micturition). The ICIQ-SF is comprised of three questions regarding frequency, severity and QoL impact of the urinary incontinence. Data are mean ± standard deviation

## Discussion

Present study findings showed that the new preoperative classification of cystocele was associated with lower urinary tract function. POP is defined as the descent of one or more structures, such as the anterior vaginal wall, posterior vaginal wall, or the apex of the vagina or vault. Our new classification system uses segments of the organ to replace previously used terms such as “cystocele, rectocele, or enterocele”, as these terms tend to suggest defects of the structures. It has been reported that POP needs to be evaluated by a standard system that is relative to the clearly defined anatomic points of a reference, such as POP-Q [6]. However, even when using this system, it was not possible to detect the accurate descent position in each structure. In a previous study, 33.8% of all POP patients were found to have anterior wall prolapse, which is the most commonly seen condition in women [7]. Therefore, anterior wall prolapse is an important issue that needs to be evaluated in POP patients [8], with level 1 support (in accordance with DeLancey’s description) needed during any surgical operation [9]. Lawrence et al. [10] reported that 44% and 37% of POP patients have SUI and overactive bladder (OAB), respectively. Furthermore, Romanzi et al. [11] reported finding a significant association between VD and POP. In addition, increasing severity of POP was reported to be weakly to moderately associated with several specific symptoms that were related to VD and urinary incontinence [12]. Based on these previous reports, patients with POP appear to experience symptoms that are not necessarily correlated with site-specific defects. Moreover, another review stated that women with high-grade POP have increased urethral closure pressure and pressure/transmission ratios, which decreases after the prolapse is reduced by surgery. Thus, the definitive aim of prolapse surgery is to reduce or eliminate the PVR in conjunction with its accompanying contribution to the VD diagnosis [3]. Advanced POP may cause changes in the voiding function, therefore resulting in a variety of urinary symptoms, such as urinary leakage and obstructive voiding [13]. These voiding difficulties have been shown to be associated with increasing severity of POP [11]. The simultaneous recording of urodynamic parameters and x-ray imaging of the lower urinary tract has shown that severe prolapse below the urethra caused obstruction due to compressing the urethra against the pubic bone [14]. However, at the present time there is no objective method that can be used to identify the position and the number of bladder hernia orifices. In our current study, we define a new clinical classification based upon the position and the number of bladder hernia orifices of cystocele determined through the use of cystoscopy. During the procedure used to inspect the bladder, patients were instructed to perform the Valsalva maneuver (Fig. 1). By utilizing this strategic procedure, this made it possible to identify the precise position of the hernia orifice such as urethra, trigone, and posterior wall type.

Chae et al. examined patients with high-stage POP and reported finding that subjects having points Aa and Ba that were significantly longer had a higher incidence of VD [15]. However, our results indicated that points Aa and Ba were uncorrelated with bladder anatomical changes. Our findings showed that only point Gh demonstrated a greater value in patients with the trigone type as compared to the posterior wall type. Gh describes the length of the genital hiatus (distance from the external meatus to the posterior fourchette). Vaughan et al. reported that having a persistently 4 cm or greater Gh after native tissue repair was associated with higher odds of having a future anatomic failure [16]. Historically, Gh was a critical pelvic valve, with Gh repairs a part of the standard POP repair procedure [17]. Delancey reported that Gh elongation could lead to a downward shift in the pelvic organs, thereby placing increased stress on both level I and II support [18]. In our current study, we found that the trigone type exhibited a more downward apical prolapse anatomically during the transvaginal inspection. This might indicate that transvaginal inspection is not completely correlated with bladder hernia. When there is weakening of the supporting tissues of the pelvic structure, this makes it difficult to maintain the anatomic position of the bladder, thereby leading to the formation of herniation called cystocele [19]. Furthermore, vaginal introitus is not a simple extension of the vaginal wall structure, but distinguishes itself independently from vaginal anatomical structures. Since the dorsal side of the bladder contains many structures, it is unlikely that all patients with cystocele would have the same defect of the vaginal wall. When POP patients are diagnosed as having transverse anterior compartment defects, they undergo site specific repairs. Thus, when performing native tissue reconstruction for site specific repairs, the detection of the specific defect is critical. In our current study, there were no data reported for each of the compartment defects that were determined during the transvaginal detailed examination. This new classification could be able to confirm the prolapse position in more detail than the previous classification by POP-Q system and provide detailed information that could be used during any of these native tissue reconstructions. And in this study the posterior wall type had statistically lower values for average urine flow rate versus the urethra and trigone type. The new classification may also make it possible to predict possible future lower urinary tract symptoms.

Data collected on the voiding functions showed that patients with posterior wall type exhibited a significantly deteriorated (increasing PVR (*P* = 0.013) and decreased urine flow rate (*P* = 0.020)) as compared to the multiple hernia orifices type. It has been previously reported that there is a significantly increased prevalence of voiding difficulty in conjunction with increasing grades of POP [20]. The major mechanism involved in these cases appears to be related to an anterior prolapse that can potentially cause mechanical bladder outlet obstruction by urethral kinking. However, in our new classification groups, not all of the anterior prolapse patients exhibited kinking of the urethra. In some of the patient groups there was single posterior wall herniation. In these cases, patient pressure transmission from the bladder to the urethra is potentially inefficient and thus, can lead to deteriorated voiding function. When VD patients were evaluated, patients with multiple hernia orifices type were found to have fair pressure transmission mechanisms as compared to those with only posterior wall herniation. A possible explanation for this finding may be that the prolapse of the whole anterior wall compartment produces more direct pressure on the urethra. However, further research will need to be undertaken in order to definitively understand this phenomenon.

The PFDI-20, which assesses the degree of discomfort caused by symptoms of pelvic floor dysfunction, consists of three parts: the POP distress, urinary distress, and colorectal-anal distress inventories [4]. Our current study found no significant differences among the four groups for the urinary distress inventory. Moreover, the lower urinary tract symptoms in the ICIQ-SF were not significantly different among the four groups. Although some reports have shown that the anterior vaginal wall prolapse was correlated with urinary incontinence, linking of the increasing rates of incontinence to the increasing stages of cystocele remains challenging [7]. Furthermore, while low-grade prolapse is often associated with urinary incontinence, patients with high-grade anterior compartment prolapse often do not complain of urinary incontinence [14]. Poor transmission of abdominal pressure to the urethra during sudden increases in intra-abdominal pressure is believed to contribute to urinary incontinence. The pubocervical fascia normally helps these components of the anterior vaginal wall to form a hammock that supports the bladder and bladder neck. In the grouping used for our current study, we could not find any significant difference for the urinary incontinence. Even in high-grade POP-Q, patients with POP exhibit a variety of prolapse positions (hernia orifice) in the bladder, various lower urinary tract dysfunctions and symptoms that occur during the progressive course of their disease. Furthermore, the combination of hernia orifice sites according to this new classification may have changed with POP deterioration, however since this study is performed retrospective style, further studies are needed.

The limitations for our current study included having only a small number of patients. Thus, this made it difficult to define the exact correlation between the new classification and the objective and subjective symptoms. A large-scale patient group will need to be evaluated in a further study in order to determine the critical role of our new classification in lower urinary tract function. Nevertheless, the results of this preliminary study suggest the importance of documenting anatomical changes of the bladder in order to improve our knowledge and clinical understanding of anterior vaginal wall prolapse.

## Conclusion

Findings for the current study demonstrate that a new subgrouping might help to more clearly identify and properly evaluate lower urinary tract function, with the results able to be used to compare between patients.

## Supplementary Information

**Additional file 1**. Detailed data of 49 patients who participated in this study.

**Additional file 2: Appendix 1**. Pelvic Floor Disability Index (PFDI-20). Measurements of the clinic condition of the pelvic floor related to the symptoms perceived by the participants.

## Data Availability

The datasets used and/or analyzed during the current study are available as Supplementary Material (Additional file 1).

## Abbreviations

SUI: Stress urinary incontinence
POP: Pelvic organ prolapse
VD: Voiding dysfunction
PVR: Postvoid residual urine volume
PFDI-20: Questionnaires pelvic floor distress inventory short form
ICIQ-SF: International consultation of incontinence questionnaire-short form
OAB: Overactive bladder
