# Supplementary material for: Novel insight into the correlation between hernia orifice of cystocele and lower urinary tract function: a pilot study
Source: BMC Womens Health. 2022 May 13;22:164. doi: 10.1186/s12905-022-01747-5 (PMC9102938; doi:10.1186/s12905-022-01747-5)
Supplement: Supplementary file 2 — Additional file 2: Appendix 1. Pelvic Floor Disability Index (PFDI-20). Measurements of the clinic condition of the pelvic floor related to the symptoms perceived by the participants. [file 12905_2022_1747_MOESM2_ESM.pdf]

Pelvic Floor Disability Index (PFDI-20)

Instructions: Please answer all of the questions in the following survey. These questions will ask you if you have certain bowel, bladder, or pelvic symptoms and, if you do, **how much they bother you**. Answer these by circling the appropriate number. While answering these questions, please consider your symptoms over the last 3 months. The PFDI-20 has 20 items and 3 scales of your symptoms. All items use the following format with a response scale from 0 to 4.

<u>Symptom scale:</u> **0 = not present**
1= not at all
2 = somewhat
3 = moderately
4 = quite a bit

Pelvic Organ prolapse Distress Inventory 6 (POPDI-6)

Do You…	**NO**	**YES**
1. Usually experience pressure in the lower abdomen?	0	1 2 3 4
2. Usually experience heaviness or dullness in the pelvic area?	0	1 2 3 4
3. Usually have a bulge or something falling out that you can see or feel in your vaginal area?	0	1 2 3 4
4. Ever have to push on the vagina or around the rectum to have or complete a bowel movement?	0	1 2 3 4
5. Usually experience a feeling of incomplete bladder emptying?	0	1 2 3 4
6. Ever have to push up on a bulge in the vaginal area with your fingers to start or complete urination?	0	1 2 3 4

Colorectal-Anal distress Inventory 8 (CRAD-8)

Do You…	**NO**	**YES**
7. Feel you need to strain too hard to have a bowel movement?	0	1 2 3 4
8. Feel you have not completely emptied your bowels at the end of a bowel movement?	0	1 2 3 4
9. Usually lose stool beyond your control if your stool is well formed?	0	1 2 3 4
10. Usually lose stool beyond your control if your stool is loose?	0	1 2 3 4
11. Usually lose gas from the rectum beyond your control?	0	1 2 3 4
12. Usually have pain when you pass your stool?	0	1 2 3 4
13. Experience a strong sense of urgency and have to rush to the bathroom to have a bowel movement?	0	1 2 3 4
14. Does part of your bowel ever pass through the rectum and bulge outside during or after a bowel movement?	0	1 2 3 4

Urinary distress Inventory 6 (UDI-6)

Do You…	**NO**	**YES**
15. Usually experience frequent urination?	0	1 2 3 4
16. Usually experience urine leakage associated with a feeling of urgency, that is, a strong sensation of needing to go to the bathroom?	0	1 2 3 4
17. Usually experience urine leakage related to coughing, sneezing or laughing?	0	1 2 3 4
18. Usually experience small amounts of urine leakage (that is, drops)?	0	1 2 3 4
19. Usually experience difficulty emptying your bladder?	0	1 2 3 4
20. Usually experience pain or discomfort in the lower abdomen or genital region?	0	1 2 3 4

Scoring the PFDI-20
Scale Scores: Obtain the mean value of all of the answered items within the corresponding scale (possible value 0 to 4) and then multiply by 25 to obtain the scale score (range 0 to 100). Missing items are dealt with by using the mean from answered items only.

PFSI-20 Summary Score: Add the scores from the 3 scales together to obtain the summary score (range 0 to 300).
